# Development of positive antinuclear antibodies and rheumatoid factor in systemic juvenile idiopathic arthritis points toward an autoimmune phenotype later in the disease course

**DOI:** 10.1186/1546-0096-12-28

**Published:** 2014-07-16

**Authors:** Boris Hügle, Claas Hinze, Elke Lainka, Nadine Fischer, Johannes-Peter Haas

**Affiliations:** 1 German Center for Pediatric and Adolescent Rheumatology, Gehfeldstrasse 24, 82467 Garmisch-Partenkirchen, Germany; 2 Department of Pediatric Rheumatology and Immunology, University Children's Hospital Münster, Münster, Germany; 3 Department of Pediatric Rheumatology, University Duisburg-Essen, Children’s Hospital, Essen, Germany

**Keywords:** Juvenile systemic arthritis, Juvenile idiopathic arthritis, Antinuclear antibodies, Rheumatoid factor - autoimmunity

## Abstract

**Background:**

Systemic juvenile idiopathic arthritis (sJIA) is commonly considered an autoinflammatory disease. However, sJIA patients may develop aggressive arthritis without systemic inflammation later in the disease, resembling an autoimmune phenotype similar to other subtypes of JIA. The objective of this study was to determine whether antinuclear antibodies (ANA) and rheumatoid factor (RF) will develop in patients with sJIA over the course of the disease.

**Findings:**

A single center sample of sJIA patients with follow-up of more than one year was obtained. A retrospective chart survey was used to extract demographic and clinical data as well as presence and titers of ANA and RF at diagnosis and during follow-up. 32 patients were included in the study, with a median age of 4.2 years and median follow-up of 6.0 years. 8/32 patients had ANA titers ≥ 1:80 at diagnosis, with 22/32 patients showing rising ANA titers with titers ≥ 1:80 at last follow-up (p =0.001). 10/32 patients had a positive RF at least once during follow-up, compared to 0/32 at diagnosis (p = 0.001). In 5/10 patients, positive RF was documented at least twice, more than twelve weeks apart. Patients treated with TNF antagonists were not significantly more likely to develop positive ANA titers (p = 0.425) or positive RF (p = 0.703).

**Conclusions:**

Patients with sJIA developed increased ANA titers and positive RF over the course of the disease, independent of treatment with TNF antagonists. This might point towards an autoimmune, rather than an autoinflammatory phenotype later in the course of sJIA.

## Findings

### Introduction

Systemic juvenile idiopathic arthritis (sJIA) is a disease characterized by marked systemic inflammation and a high rate of severe and potentially life-threatening manifestations. While categorized as a subtype of juvenile idiopathic arthritis (JIA) according to the ILAR-criteria, sJIA is currently considered to represent an autoinflammatory rather than an autoimmune syndrome [1-3]. Autoinflammatory conditions are thought to represent abnormalities of the innate immune system with hallmark findings of seemingly unprovoked inflammation, in contrast to autoimmune conditions caused by autoreactive T or B lymphocytes and autoantibodies. This might be an oversimplification, since features of both autoinflammation and autoimmunity are typically present in most conditions; hence, a classification of disorders along an axis between autoinflammation and autoimmunity has been proposed [2,4].

Although in sJIA, systemic inflammation tends to decrease over time in most patients, approximately half of sJIA patients can be expected to develop an aggressive polyarthritis [5]. This course of sJIA leads to a phenotype of chronic polyarthritis similar to that observed in other forms of JIA in which autoimmunity appears to play an important role.

The objective of this study was to determine frequencies of ANA and RF as circumstantial markers for autoimmunity in patients with sJIA over the course of the disease.

### Methods

Patient sera and clinical data were acquired from the AID-Net database ( http://www.aid-register.de), a German registry and biobank that prospectively collects information and biomaterials of patients with autoinflammatory syndromes including periodic fevers syndromes and sJIA [6]. A single center sample of all patients with sJIA at the German Center for Pediatric and Adolescent Rheumatology was screened between January 2010 and July 2012, and all sJIA patients with a follow-up of more than one year were included. A retrospective chart survey was used to extract demographic data, clinical course including total joint count and treatment as well as presence and titers of antinuclear antibodies (ANA) and rheumatoid factor (RF) at beginning and during follow-up. All ANA and RF studies were performed in a single laboratory to ensure comparability, and the laboratory methods were used consistently during the follow-up period. ANA titers were determined using the HEp-2000 fluorescent ANA-Ro test system (Immuno Concepts, Sacramento, USA), and rheumatoid factors were determined using the Rheuatoid Factors II test kit with a cobas c 311 analyzer (Roche Diagnostics GmbH, Mannheim, Germany). Analysis was performed using descriptive statistics, Student’s T-Test/Fischer’s Exact test, one-way ANOVA (ANA-positive, ANA-negative patients and ANA-converted patients), and Spearman’s correlation (ANA-titers and total active joint count). Statistical analysis was performed with SPSS version 21.0 (SPSS Inc., Chicago, USA).

### Results

**Figure 1:**
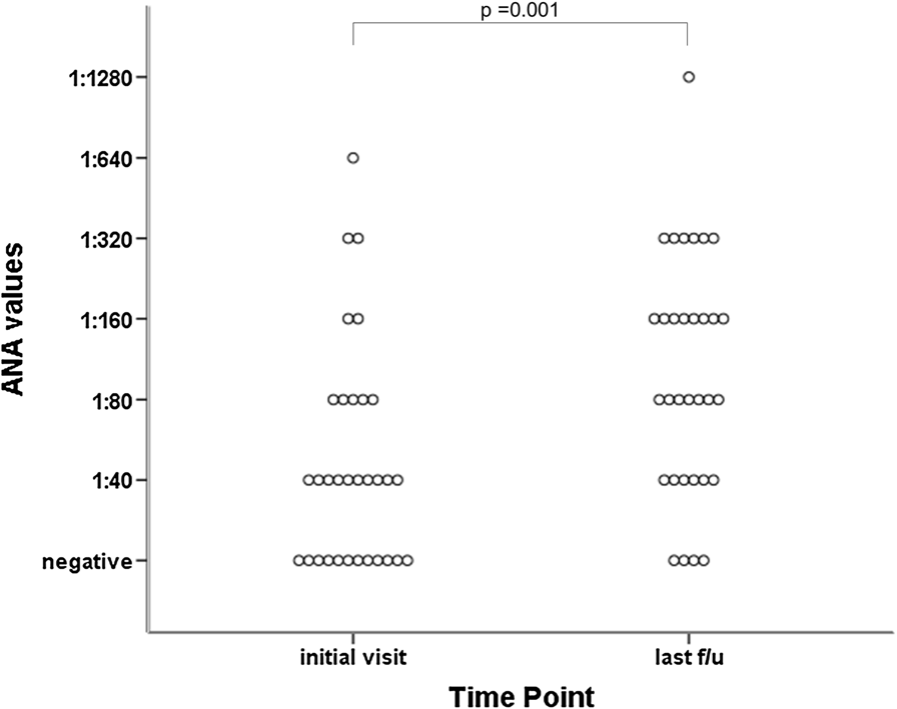
Dot dispersion plot of antinuclear antibodies titers at diagnosis and at last follow-up in patients with systemic juvenile idiopathic arthritis (n = 32).

32 patients were included in the study (20 of these female), with a median age at diagnosis of 4.2 years (range 0.5 – 11.4 years). The median follow-up was 6.0 years (range 1.1 – 17.3 years). During the course of disease, 96.8% were treated with disease-modifying antirheumatic drugs (of those: methotrexate 100%, azathioprine 52% and cyclosporine A 48%), 65.6% with any TNF antagonist (of those: etanercept 100%, infliximab 14% and adalimumab 29%), 65.6% with anti-interleukin(IL)-1 antagonists and 15.6% with anti-IL-6 antagonists. 8/32 patients had ANA titers ≥ 1:80 at diagnosis, with 22/32 patients showing a titer of ≥ 1:80 at last follow-up (p =0.001) (Figure 1). There was no difference according to age at diagnosis (p = 0.949), length of follow-up (p = 0.197), maximum joint count at any time (p = 0.348) or total joint count at last follow-up (p = 0.314) among persistently ANA-negative, persistently ANA-positive and ANA-seroconverted patients. Using measures at 463 time points, there was no correlation between ANA titers and total active joint count (r = 0.180, p = 0.703). During follow-up, 10/32 patients had a positive RF at least once, compared to 0/32 at diagnosis (p = 0.001). In five of these patients, positive RF were documented at least twice, more than twelve weeks apart. Patients treated with TNF antagonists were not significantly more likely to develop a positive ANA (p = 0.425) or positive RF (p = 0.703).

### Discussion

This retrospective, descriptive single-center study shows an increasing frequency of positive ANA and RF titers over time in patients with sJIA. In earlier studies, various types of autoantibodies, including ANA and RF, have been found in up to one third of patients with sJIA [7-10]. However, these studies did not differentiate between varying stages of the disease. While ANA are found in healthy children and tend to increase with age, the frequencies of autoantibodies demonstrated in our study are certainly beyond those found in normal controls [11,12]. A correlation between ANA titers and arthritis could not be demonstrated. While TNF blockade can lead to development of ANA, our analysis shows that this is most likely not a factor in our patient population [13].

As has been previously observed, patients with sJIA can be divided into three clinical phenotypes, with either mono- or polyphasic course or persistent disease [5]. The clinical symptoms tend to change over time. Most patients with persistent disease show no systemic signs at 6 months after diagnosis but instead exhibit a particularly severe progressive polyarthritis, often involving the hip and shoulder joints. The immunopathogenic mechanisms in the inflamed joints of patients with sJIA are poorly understood. In other forms of JIA (e.g. oligo-, poly- and spondyloarthritis) both indicators of innate immunity, such as elevated myeloid-related proteins (MRP) 8 and 14 produced by neutrophils and infiltration by neutrophils, monocytes and macrophages, and indicators of adaptive immunity, such as prominent CD4 lymphocyte infiltration, are found directly in the synovium and synovial fluid [14,15]. To our knowledge, such detailed data are not available for patients with sJIA, and the reason for the particularly aggressive polyarthritis remains enigmatic.

**Figure 2:**
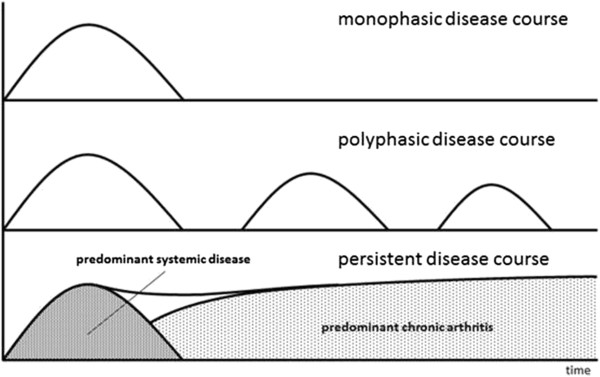
**Concept schematic of the different disease courses of systemic juvenile idiopathic arthritis.** The lower curve shows the postulated two phases of persistent disease course, with initially predominant systemic disease and development of chronic polyarthritis later in the disease course.

The prodromal, systemic phase of sJIA appears to correlate well with a massive activation of the innate immune system, as indicated e.g. by PBMC gene expression studies showing marked upregulation and/or overrepresentation of innate immune signaling pathways, downregulation of adaptive immunity signaling pathways, and extremely elevated MRP8/14 levels [16,17]. Taking into account the clinical picture, the lack of female preponderance, the lack of HLA class II associations, the general lack of autoantibodies in this disease phase and an excellent response to IL-1 and IL-6 blockade, sJIA may well be regarded as a 'pure’ autoinflammatory syndrome. This leads to the question: How and why is there, in a substantial proportion of patients, a transition to an aggressive polyarthritis, often with cessation of the extreme systemic inflammation initially observed? The presence of increasing autoantibody titers demonstrated here supports the hypothesis that the initial inflammatory phenotype of sJIA might induce B- and T-cell mediated autoimmunity later in the disease, for example via downstream activation of antigen presentation. Figure 2 illustrates this postulated mechanism of a two-phased disease course, with initial systemic disease driven by autoinflammatory processes, and ongoing arthritis caused by autoimmunity. Whether or not this observation is pathogenically relevant or merely an epiphenomenon representative of nonspecific polyclonal activation is unclear and cannot be answered by this study.

An additional argument for a shift in pathogenesis is the observation that in a substantial proportion of sJIA patients anti IL-1 treatment does not control the arthritis, or the response to treatment with anti-IL1 agents decreases over time [18-20]. On the other hand, it has been hypothesized that treatment with IL-1 antagonists in the early (i.e. predominantly autoinflammatory) phase might even prevent transition into a course characterized by refractory polyarthritis [21]. Blocking the IL-1 pathway might therefore have a function in suppressing the transition to persistent B- and T-cell activation.

The assumed transition from an autoinflammatory to an autoimmune phenotype should be accompanied by a marked switch in the types of expressed genes, from a pattern with predominantly innate immune pathway activation towards a more T-cell driven picture. Gene expression profiles in peripheral blood mononuclear cells (PBMC) of new onset sJIA show up-regulation of genes from the innate, and down-regulation of genes from the adaptive immune system [22]. In particular, compared to other subtypes, new-onset sJIA shows down-regulation of natural killer cell, T cell, and antigen-presentation pathways [16]. Clustering of expressed genes can differentiate between patients according to active and inactive disease, independent of their medications [23]. Similarly, cluster analysis can distinguish between patients with high and low ferritin levels, corresponding to the level of systemic inflammation [24]. However, to date gene expression analysis has been limited to either new-onset sJIA or patients in remission. It would be interesting to see if sJIA patients with arthritis and no systemic features, later in the disease course, show a gene expression profile closer to patients with polyarticular JIA, i.e. elevated expression of monocyte markers and transforming growth factor β-inducible genes [25].

Strengths of our study include that all consecutive patients within a single center were included in this study, thus representing a rather homogeneous patient population. Weaknesses of our single-center study include its retrospective nature, the lack of precise identification of the progressive polyarthritis subtype reported above, and the lack of measuring other, non-specific biomarkers of chronic immune activation (e.g. erythrocyte sedimentation rate or IgG levels); since our center is a tertiary referral center, there may have been a bias towards more serious cases. It should be noted that many of the results in this study are based on single instances of elevated test results. Both ANA and RF can be unspecifically elevated due to infections and other causes of immune activation, introducing possible causes of error. Especially with rheumatoid factor, ILAR guidelines recommend the determination of two elevated titers 3 months apart, which could not be confirmed in each patient in this study [3]. However, individual patients in our study trended toward increasing titers over time. ANA titers might also be affected by medications beyond TNF inhibitors, as well as immunosuppression induced by these medications. The retrospective nature of this study, as well as the variety of medications used in this study, precluded any further analysis.

### Conclusion

In summary, this study demonstrates increasing serum autoantibody titers over time in a proportion of patients with sJIA, indicating the potential role of lymphocyte activation and autoimmune processes in this primarily autoinflammatory condition. It remains unclear if this is indeed of pathogenic relevance, or merely an epiphenomenon. Further studies, especially longitudinal gene expression studies in target tissues, such as synovium or synovial fluid, in patients with the chronic polyarthritis phenotype of sJIA, will be necessary to elucidate this phenomenon.

## Competing interests

The authors declare that they have no relevant conflict of interests.

## Authors’ contributions

BH and JPH conceived of the study, BH, CH and JPH drafted the manuscript. CH and NF participated in data collection and reviewed the manuscript. EL participated in researching and drafting the manuscript. All authors read and approved the final manuscript.
